# A Phase-Dependent Model of Sorghum (*Sorghum bicolor*) Cold Acclimation: Integrating Multi-Layered Networks and Alternative Splicing Signatures

**DOI:** 10.3390/biology15070560

**Published:** 2026-03-31

**Authors:** Firat Kurt

**Affiliations:** Department of Plant Production and Technologies, Faculty of Applied Sciences, Muş Alparslan University, 49250 Muş, Türkiye; firat.kurt.usa@gmail.com

**Keywords:** sorghum, cold stress, RNA-Seq, transcription factors, regulators, kinases, alternative splicing, epigenetics

## Abstract

Cold stress limits sorghum productivity, yet the temporal organization of its molecular response remains incompletely understood. In this study, a multi-layered transcriptomic approach was employed to analyze the cold response of sorghum across 6 h, 12 h, and 24 h. By integrating differential expression, weighted gene co-expression network analysis (WGCNA), and alternative splicing (AS) profiling, a phase-dependent regulatory model was proposed. Quantitatively, the network was initially resolved into 17 co-expression modules, which were subsequently consolidated into 10 final modules. A core set of 147 transcription factors (predominantly AP2/ERF and NAC families) was consistently associated with the response across time points. During the early shock phase (6 h), the broad repression of energy-associated transcripts suggests rapid intracellular stabilization. The transition phase (12 h) was characterized by transcriptomic shifts suggestive of chromatin-level regulation and post-transcriptional adjustments. By late acclimation (24 h), the reorganization of stress-associated modules indicates a progression toward a stabilized regulatory state. Furthermore, the identification of dynamic AS events across multiple regulatory families suggests that isoform diversification is a crucial parallel regulatory layer. Moving beyond static expression profiling, this study provides a comprehensive temporal framework of sorghum cold acclimation and identifies phase-specific candidate genes for future experimental validation in C4 crops.

## Simple Summary

Sorghum is a globally important cereal crop known for its high drought tolerance; however, its sensitivity to cold temperatures significantly limits its cultivation. To better understand the timeline of its molecular response, this study computationally analyzed transcriptomic data from sorghum plants exposed to cold stress at 6, 12, and 24 h. Rather than focusing solely on individual differentially expressed genes, bioinformatic approaches were used to infer gene interaction networks and examine alternative splicing patterns (variations in how RNA messages are processed). The findings suggest that the plant response unfolds in distinct phases: an initial “shock” phase marked by reduced expression of energy-associated genes, a “transition” phase associated with core regulatory activity, and a late “acclimation” phase in which the transcriptional response appears more stabilized. By modeling these temporal changes, this study proposes a phase-dependent framework and highlights a prioritized set of candidate genes. These results provide a data-driven basis for future experimental studies aimed at understanding cold resilience in sorghum.

## 1. Introduction

Sorghum (*Sorghum bicolor*), a C4 plant species [1], is the fifth most widely cultivated crop worldwide [2]. Owing to its lower water requirements compared with other cereal crops [3], sorghum serves as a staple food source for more than 500 million people, particularly in hot and arid regions of Sub-Saharan Africa and Asia where maize often fails to thrive [4]. However, sorghum lacks the advanced cold acclimation mechanisms observed in cool-season cereals (e.g., winter wheat and barley), and exhibits limited tolerance to low temperatures, similar to rice and maize [5]. While the physiological symptoms of cold sensitivity, such as reduced germination and chlorophyll degradation, are well-documented, the regulatory architecture governing its adaptive responses remains insufficiently understood, limiting crop improvement in temperate regions.

Cold stress perception in sorghum is initiated by changes in plasma membrane fluidity and specific sensors such as *COLD1* [6]. Following perception, secondary messengers such as reactive oxygen species (ROS) and inositol trisphosphate (*IP*_3_) activate protein kinase cascades [7]. Recent evidence highlights the role of the sorghum cyclic nucleotide-gated channel (*SbCNGCs*) family—particularly *SbCNGC1*—as core Ca^2+^ channels initiating these signaling events [8]. Although studies in rice have demonstrated that SnRK2 kinases regulate Ca^2+^ influx via CNGC channels [8,9], the integration of these signaling components within a coordinated sorghum regulatory network remains unclear.

At the transcriptional level, the cold response is largely driven by the ICE1-CBF/DREB transcriptional regulatory cascade. Transcription factors such as *ICE1* and *CAMTA* activate CBF/DREB genes, which subsequently induce cold-regulated (COR) genes [6,10] to maintain cellular homeostasis via the accumulation of osmoprotectants such as proline, sugar alcohols (ribitol and sorbitol), and anthocyanins [11]. In the sorghum genome, the AP2/ERF family plays a central role; for instance, cold-induced expression of *SbERF027* [9] and an *AtCBF3-like* transcription factor, *SbCBF6*, exhibit higher expression levels in cold-tolerant accessions [6,10]. Additional regulators, including MYB62, NAC1, and various WRKY factors, also contribute to this complex transcriptional network [6,12].

Despite the identification of these individual regulators, the temporal coordination and multilayered control of cold stress responses in sorghum remain poorly understood [5]. Previous studies have largely relied on static transcriptomic analyses, leaving a significant knowledge gap regarding how these responses are organized at the network level and modulated by post-transcriptional mechanisms like alternative splicing (AS) [10,13]. To address this gap, the present study applies an integrated systems biology framework combining differential gene expression analysis, Weighted Gene Co-expression Network Analysis (WGCNA), and alternative splicing (AS) profiling. The selection of 6, 12, and 24 h time points was strategically designed to capture three distinct functional phases: the acute shock phase (6 h), the transitional phase (12 h), and the late acclimation phase (24 h). By integrating these temporal dynamics, this study aims to identify key regulatory hubs and characterize the network-level organization of cold stress responses in sorghum.

## 2. Materials and Methods

### 2.1. Data Acquisition

This study investigated the transcriptomic responses of sorghum under cold stress by re-analyzing a subset of a publicly available RNA-seq dataset. The dataset used in the study corresponds to the cold stress subset of the accession number PRJNA1159475 available in the National Center for Biotechnology Information (NCBI) Sequence Read Archive (SRA) database (https://www.ncbi.nlm.nih.gov/sra; retrieved on 26 January 2026) [14]. To clarify the biological design of the original experiment, as reported by the data generators, the sorghum genotype *Sorghum bicolor* L. cv. Hwanggeumchal was utilized. In the original experiment, seeds were surface-sterilized and grown in soil under controlled environmental chamber conditions (16-h photoperiod, 25/23 °C day/night temperature). Two-week-old seedlings were then subjected to acute cold stress at 4 °C. Within this subset, raw RNA-Seq reads obtained from leaf tissue samples of sorghum seedlings exposed to cold stress at different time points (control group, and 6, 12, and 24 h) (comprising three biological replicates per condition and time point) were downloaded and incorporated into the bioinformatics analysis workflow.

#### 2.1.1. Data Extraction and Quality Control

All data analysis and processing steps were performed on the Galaxy platform [15]. Raw reads were downloaded from the NCBI SRA database in FASTQ format using the Faster Download and Extract Reads in FASTQ tool in Galaxy (v.3.1.1) [14]. To improve the efficiency of raw data processing and to assess read quality, a rapid preliminary quality check was performed using the Falco tool (v1.2.4) [16]. Quality metrics for all samples were combined using the MultiQC tool (v1.33), and comprehensive quality control reports were generated [17].

The analyses showed that the 101-base-pair (bp) paired-end reads exhibited average Phred quality scores above the Q30 threshold at all time points, and above Q35 in the 24-h samples. Adapter contamination and ambiguous base (N-content) levels were found to be negligible (<0.1%). A consistent bimodal distribution was observed in the per-sequence GC content analysis, with peaks at approximately 46% and 68%. Together with the high read duplication rates, this pattern was interpreted as a biological signal rather than a technical artifact. This likely reflects transcripts originating from organelle genomes (chloroplasts and mitochondria) and from highly expressed genes involved in stress responses (e.g., housekeeping and stress-responsive genes). Based on these results, the raw read data were preprocessed using the fastp tool (v1.0.1) [18]. In this vein, automatic adapter detection was enabled, and the analysis sampling size for overrepresented sequences was set to 20. During quality filtering, a minimum Phred Q20 threshold was applied, and reads shorter than 30 bp after trimming were excluded from further analysis. In addition, PolyG tail trimming was enabled with a minimum length of 10 nucleotides, while the low-complexity filter and polyX tail trimming were disabled. All other parameters were kept at their default settings.

#### 2.1.2. Post-Filtering Data Status

Following quality control of the raw data obtained from the 6, 12, and 24-h cold stress treatments and their corresponding control groups, a high data retention rate ranging from 98.5% to 99.2% was achieved across all time points. When examined on a group basis, an average of 60.1 and 66.7 million clean reads were obtained from the control and stress samples at 6 h (GC content: 53.9% and 52.1%, respectively); 64.7 and 61.3 million reads at 12 h (GC content: 54.3% and 52.4%); and 60.1 and 58.8 million reads at 24 h (GC content: 53.9% and 53.3%). In all samples, Q30 values—reflecting base-calling accuracy—met standard analytical thresholds (~99.9%), and adapter contamination was negligible (<0.5%). The data that successfully passed quality control were subsequently used for alignment to the *Sorghum bicolor* NCBI v3 reference genome and for differential gene expression analyses.

#### 2.1.3. Reference Genome and Gene Annotation

For RNA-Seq read alignment and gene expression analysis, the *Sorghum bicolor* NCBI v3 reference genome was used. The corresponding genome sequence file and gene annotation file were obtained from the Ensembl Plants database (Release 60) [19,20].

#### 2.1.4. Validation of Library Strandness

To determine the strand specificity of the RNA-Seq dataset, the sorghum reference gene annotation file (GTF) was converted to BED12 format on the Galaxy platform using the UCSC GTF-to-BED12 converter [21]. Subsequently, read orientation was analyzed using the Infer Experiment tool from the RSeQC package (v5.0.3) [22]. The results indicated that approximately 97% of the reads matched the “1+-, 1-+, 2++, 2--“ configuration. These findings confirm that the libraries are strand-specific and that sequencing was performed in the reverse-stranded (fr-firststrand) mode.

#### 2.1.5. Alignment to the Sorghum Genome

Raw reads from the control and cold stress treatments at 6, 12, and 24 h were mapped to the reference genome using the STAR algorithm [23]. As a result of the analyses, the overall mapping rates in the control groups were recorded as 88.5%, 86.2%, and 83.3% at the respective time points, while the corresponding rates in the cold stress groups were 84.7%, 85.0%, and 77.9%. The majority of the mapped reads were aligned uniquely to a single locus in the reference genome. These uniquely mapped read proportions were 86.7%, 84.5%, and 81.5% for the control groups, and 83.4%, 83.6%, and 76.6% for the stress-treated groups, respectively. Across all time points and conditions, a consistently low mismatch rate of approximately 0.3% further confirmed the high quality of both the sequencing and alignment processes.

#### 2.1.6. Gene Read Count Quantification

The results obtained from the FeatureCounts analysis (v2.1.1) [24] confirmed adequate mapping quality and assignment efficiency for transcriptomic profiling across all time points (6, 12, and 24 h). Early-stage (6-h) samples exhibited the highest consistency, with all replicates in both control and stress groups remaining above the acceptable threshold (>75%), showing assignment rates of 81.6%, 82.3%, and 80.5% for the control group, and 75.7%, 82.3%, and 75.1% for the stress group. At 12 h, assignment rates in the cold stress group reached up to 87.4% (control: 71.3–81.0%; stress: 75.2–87.4%), maintaining robust reliability. In contrast, samples representing prolonged exposure at 24 h showed a moderate increase in inter-sample variation (control: 63.4–73.9%; stress: 68.0–81.7%). Nevertheless, the overall sequencing depth and low proportion of unassigned reads observed at all time points demonstrate the specificity of the library preparation process and confirm the suitability of the dataset for subsequent differential expression analyses.

#### 2.1.7. Differential Gene Expression Analysis (DEGs)

Differential gene expression analyses between the stress and control groups at each time point (6, 12, and 24 h) were performed using the gene count data obtained with FeatureCounts and the DESeq2 package (v2.11.40.8) [25]. To accurately assess the treatment effect at each specific time point, the DESeq2 design formula~condition (cold stress vs. control) was applied. Genes were defined as significantly differentially expressed (DEGs) based on strict statistical thresholds: an adjusted *p*-value (padj) < 0.01 and an absolute log2 fold change (|log2FC|) > 1. Overall, high correlation was observed among biological replicates, while transcriptomic divergence between the stress and control groups became increasingly pronounced with longer cold exposure durations. In this connection, Principal Component Analysis (PCA) revealed that the first principal component (PC1), which explains the largest proportion of variance, reached 88% at 12 h and 92% at 24 h, highlighting the dramatic separation between the groups. DESeq2 diagnostic plots confirmed the suitability of the data for a negative binomial distribution (dispersion estimates) and demonstrated the absence of systematic bias after normalization, as evidenced by symmetric MA plots. In addition, the strong accumulation of *p*-values near zero (anti-conservative peak) observed in the *p*-value histograms across all time points supports the presence of a substantial number of statistically significant differentially expressed genes (DEGs) in response to cold stress.

#### 2.1.8. Heatmap Visualization of Genes

To visualize gene expression patterns, raw read counts were first normalized using the Variance Stabilizing Transformation (VST) method implemented in the DESeq2 package (v2.11.40.8) [25]. Before generating the heatmap, VST values for each gene were transformed into Z-scores across samples. This step ensured that the visualization reflected relative expression differences among samples rather than absolute expression values. Heatmaps were generated in the R environment using the ComplexHeatmap package (v2.2.22.0) [26].

### 2.2. ID Mapping of Sorghum Genome Annotation Data

In this study, gene annotation data of the NCBI v3 genome assembly (GCA_000003195.3) were downloaded in GFF3 format from the Phytozome database (v3.1.1) [27] (https://phytozome-next.jgi.doe.gov/, accessed on 16 December 2025) and the Ensembl Plants database [28] (https://plants.ensembl.org, retrieved 16 December 2025). After confirming the consistency of the physical coordinates of gene models between the two databases, coordinate-based cross-mapping (chromosome, start, end) was performed using a custom script developed in the R environment [29] within RStudio [30]. Through this approach, a conversion file enabling direct correspondence between Sobic (Phytozome) and SORBI (Ensembl) gene identifiers was generated. The accuracy and integrity of the resulting ID mapping table were validated by comparison with up-to-date synonym gene lists provided via the SorghumBase database (https://sorghumbase.org, retrieved 16 December 2025) API interface [31], thereby ensuring data consistency throughout the analyses. In addition, gene lists for all transcription factors (TFs), regulators (TRs), and protein kinase families (PKs) used in this study were obtained from the plant-specific iTAK database [32] (http://itak.feilab.net/cgi-bin/itak/db_browse.cgi, retrieved 10 December 2025) and integrated with the generated ID mapping table, allowing TF, TR, and PK-related annotations to be incorporated into the dataset. Furthermore, functional annotations and family classifications for TFs, TRs, and PKs were primarily based on the iTAK database, which provides a specialized classification for plant species. To further validate these functional assignments, SorghumBase (https://www.sorghumbase.org, retrieved 5 January 2026), UniProt (https://www.uniprot.org, retrieved 5 January 2026), [33], and Phytozome databases were utilized for manual curation of key regulatory genes using the NCBI BLAST+ v2.16.0 suite (integrated into the Phytozome v13 platform) between December 2025 and January 2026. Additionally, to provide robust, sequence-level structural evidence for the highlighted hub genes and cold-related genes, the Ensembl BioMart data mining tool was employed [20,34]. Specific Transcript IDs, InterPro IDs, and their corresponding short and long functional domain descriptions were systematically extracted and integrated into the supplementary datasets (Supplementary Materials Tables S2 and S5). This approach ensured that functional naming remained consistent with plant-specific regulatory mechanisms, even in cases where general protein databases (e.g., UniProt) provided broader domain-based descriptions.

To improve clarity in gene identification and interpretation, a consistent naming approach was applied to key regulatory genes. Genes from the same family or with high sequence similarity were distinguished using numerical suffixes (e.g., SbIWS1-1, SbIWS1-2). When the iTAK database provided broad family labels (such as “Others”), additional manual annotation was performed by examining conserved domains using UniProt and SorghumBase. This allowed more specific domain-based names to be assigned (e.g., *SbSANTA*, *SbRR*, *SbBbox*). The same naming system was used consistently throughout the manuscript to ensure clear tracking of each gene across different co-expression modules and time points.

### 2.3. WGCNA of Cold-Exposed Sorghum Data

All statistical analyses and data visualizations were performed in the R statistical computing environment [29,35] using the RStudio integrated development environment [30]. In addition, gene co-expression networks were constructed and functional modules were identified from the transcriptomic data using the WGCNA package (v1.73) in R [35].

#### 2.3.1. Data Preprocessing

Prior to WGCNA, the raw data were subjected to variance stabilization using the DESeq2 package (via the varianceStabilizingTransformation, VST, method), and the resulting normalized data were merged to generate an analysis-ready dataset [35,36]. To minimize noise, eliminate uninformative background transcripts that can distort scale-free topology, and optimize computational efficiency, the lowest 10% of genes based on variance were excluded from the dataset. The remaining high-variance genes (more than 3000) were retained for network construction. Missing values (NA) were imputed using gene-wise mean values; this step is a standard requirement for WGCNA to prevent mathematical artifacts during the calculation of the correlation matrix. Additionally, sample-level outlier detection was performed using hierarchical clustering.

#### 2.3.2. Network Construction and Module Detection

Prior to network construction, missing values were imputed using gene-wise means. This standard approach was selected to maintain network stability and preserve the underlying correlation structure across the time-course dataset with minimal missingness. A signed network type was selected for the construction of the gene–gene relationship matrix. Based on the scale-free topology criterion, the soft-thresholding power that provided the best fit to the network structure was determined to be 18 (Signed R^2^ ≈ 0.78) (Supplementary Materials Figure S1). This specific power was chosen to balance the trade-off between achieving an acceptable scale-free topology fit and maintaining sufficient mean connectivity required for biological relevance. The adjacency matrix was then transformed into a topological overlap matrix (TOM) to quantify the strength of connections between genes. For module detection, average linkage hierarchical clustering combined with the Dynamic Tree Cut algorithm was applied (parameters: deepSplit = 2, minClusterSize = 30). To merge modules exhibiting similar expression profiles, correlations between module eigengenes (MEs) were calculated, and a merging cut height of 0.25 was applied. As a result of this procedure, a total of 17 initial co-expression modules were identified using the Dynamic Tree Cut algorithm, which were subsequently merged based on eigengene similarity (cut height = 0.25) into 10 final modules (Supplementary Materials Figure S2).

#### 2.3.3. Network Visualization and Export

The relationships among modules and gene intersections were visualized using the VennDiagram [37] and UpSetR packages (v1.4.0) [38]. Sample-wise expression patterns of hub genes were presented as heatmaps. For detailed analysis of network topology, edge and node lists were exported to Cytoscape (version 3.10.4) [39]. To manage global network complexity and visualize only the strongest connections, stringent filtering was applied using a quantile-based threshold of 0.999 for edge weights in the initial broad networks. However, to construct functionally integrated sub-networks (such as downstream regulatory networks) and capture biologically essential inter-modular “bridge” connections, edge weight thresholds were specifically adjusted (specifically, retaining Topological Overlap Matrix (TOM) edges > 0.5 for these sub-networks). This refined, phase-specific filtering allowed for the retention of key transcription factors and kinases that coordinate cold acclimation but would otherwise be lost under ultra-stringent global filtering.

### 2.4. Differential Alternative Splicing Analysis Using rMATS

Alternative splicing analysis was performed using rMATS v4.1.2 [40]. STAR-aligned BAM files were provided as inputs, and five types of splicing events were evaluated: skipped exon (SE), retained intron (RI), alternative 5′ splice site (A5SS), alternative 3′ splice site (A3SS), and mutually exclusive exons (MXE). Differential splicing statistics were extracted from JCEC-based output files, and significant events were filtered using thresholds of FDR ≤ 0.05 and |ΔPSI| ≥ 0.10. Shared and time-specific splicing events were identified and processed in the R environment using the dplyr (v1.1.4) [41] and UpSetR (v1.4.0) packages [38]. For the validation of key regulatory isoforms, representative splicing events and genomic read distributions were visualized using Sashimi plots generated within the Integrative Genomics Viewer (IGV v2.19.5) [42].

### 2.5. Multilayer Data Integration and Construction of the Master Annotation Matrix

To integrate multiple omics layers into a single framework, a comprehensive data integration protocol was implemented in the R environment. The process was anchored by the Node Attribute File from WGCNA (containing module memberships and connectivity) and integrated with DESeq2 results across all time points. To ensure biological robustness, only genes meeting stringent criteria (padj < 0.01 and |log_2_FC| ≥ 1) were included. To capture post-transcriptional dynamics, differential alternative splicing (DAS) events and their corresponding |ΔPSI| values from rMATS were mapped to the dataset. Finally, TFs, TRs, and PKs from the iTAK database were incorporated via gene identifiers to finalize the Master Annotation Matrix.

### 2.6. Identification of Core Regulatory Components Through Time-Resolved Intersection and Sequential Filtering

To resolve the temporal dynamics of the cold stress response, distinct analytical strategies were applied to TFs, TRs, and PKs: TFs were analyzed via an intersection approach to capture the rapid transitions and time-specific shifts in the transcriptional landscape, whereas a sequential filtering protocol was applied to TRs and PKs to isolate a stable, high-confidence signaling backbone that persists throughout the entire duration of cold exposure.

- Transcription Factors (TFs): Differentially expressed TFs were independently filtered for each time point (padj < 0.01) and processed using the InteractiVenn tool [43] (https://www.interactivenn.net, retrieved on 11 December 2025). This isolated time-specific and core (shared) TF sets. To map the molecular transitions between acute shock and acclimation, Gene Ontology (GO) enrichment analysis was performed specifically for these TF clusters using the ShinyGO (https://bioinformatics.sdstate.edu/go/, retrieved on 18 December 2025) web-based platform (v0.77) [44]. To ensure statistical rigor, the entire *Sorghum bicolor* annotated gene set was employed as the background gene universe. Significantly enriched GO terms, with a primary focus on Biological Process (BP) categories, were identified using a False Discovery Rate (FDR) significance threshold of <0.05.
- TRs and PKs: To identify the robust signaling backbone, TRs and PKs were subjected to a sequential filtering protocol (6 h → 12 h → 24 h). Only those maintaining significant expression throughout all time points were retained. Given the highly refined nature of this set, individual functional characterization and manual curation were preferred over GO enrichment to ensure a more precise biological interpretation.

### 2.7. Identification of Functional Regulatory Hubs via Network Integration

Topological hub genes were defined as network-centric nodes exhibiting the highest intra-modular connectivity (kME) as determined by the WGCNA algorithm. These genes represent the absolute mathematical centers of their respective modules and are distinct from module-specific hubs (intra-module hubs or functional regulatory hubs), which were further refined based on biological relevance and temporal expression patterns. In the final stage, the functionally defined gene sets were integrated with WGCNA topology to identify “functional regulatory hubs”. Unlike standard network hub genes—which are identified solely based on connectivity—these regulators were selected through a dual-validation approach. Candidate genes were required to meet objective operational criteria: (i) exhibit significant differential expression (padj < 0.01 and |log_2FC| > 1) at their respective time points, and (ii) possess a high module membership score, strictly defined as kME ≥ 0.8, within their assigned stress-responsive modules.

## 3. Results

### 3.1. Temporal Dynamics of Transcription Factors Responding to Cold Stress

**Figure 1:**
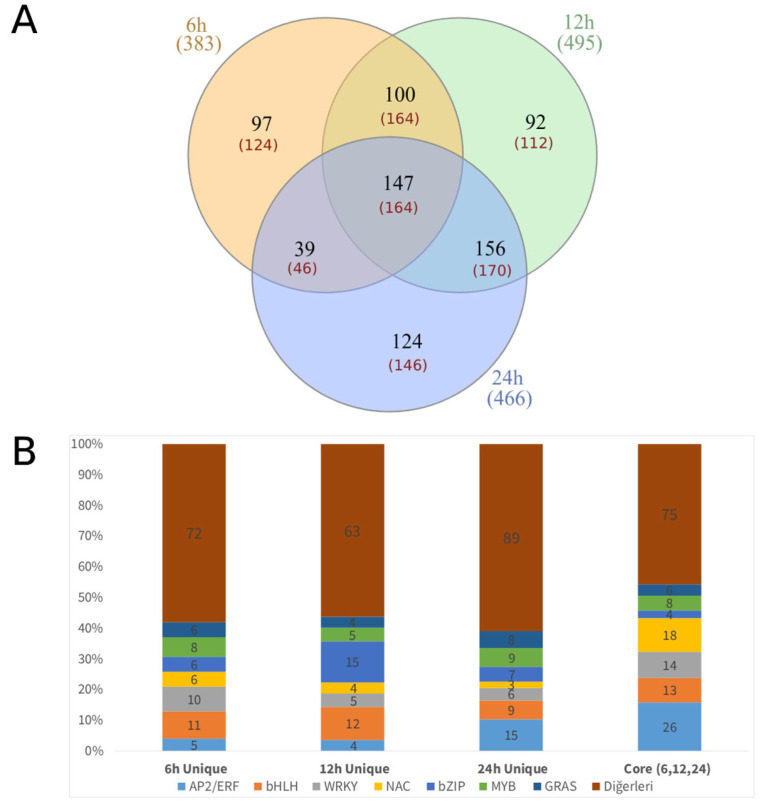
Temporal dynamics and composition of the cold-responsive transcription factors (TFs) in sorghum. Only TF families with the highest number of members are shown. A representative list of differentially regulated TFs and their family assignments is provided in Supplementary Materials Table S1. (**A**) Overlap analysis of differentially regulated TFs: Venn diagram illustrating the distribution of TF genes at 6, 12, and 24 h under cold stress (padj < 0.01). Values represent gene counts, with corresponding transcript counts provided in parentheses. (**B**) Proportional distribution of time-specific and core TF families: Stacked bar charts showing the percentage contribution of major TF families within unique (time-point specific) and core (shared across all time points) groups. The Core TF set is characterized by the dominance of AP2/ERF and NAC families, while the 12 h unique response is defined by a significant expansion of the bZIP family.

Cold stress induced distinct time-dependent changes in transcription factor (TF) expression (Figure 1). The transcriptional response began at 6 h, peaked at 12 h, and remained significantly active at 24 h, suggesting a continued regulatory effort rather than a short-term shock. Among the 147 core TF genes (164 transcripts) shared across all time points, the AP2/ERF-ERF and NAC families emerged as the primary components of the cold tolerance machinery (Supplementary Materials Tables S1 and S3).

### 3.2. GO Enrichment Analysis of TFs in Cold Stress Responses

**Figure 2:**
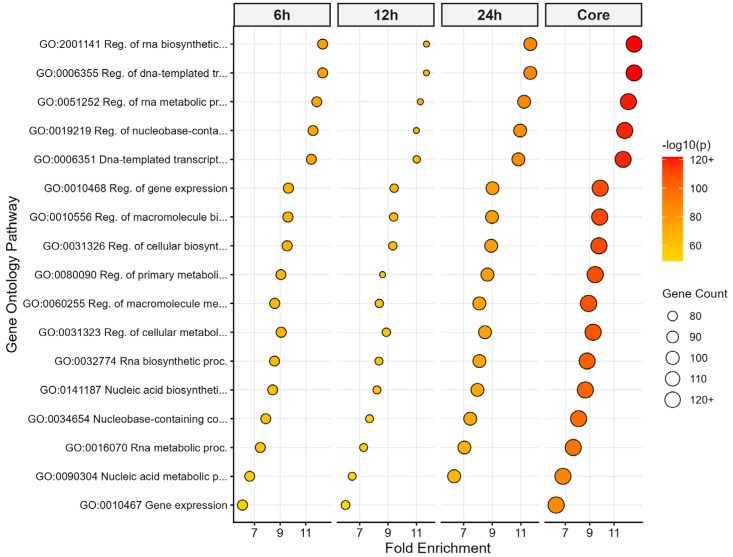
GO enrichment analysis of transcription factors involved in cold stress responses at 6 h, 12 h, 24 h, and for the TF set located at the intersection of these time points.

To evaluate the temporal behavior of transcriptional regulators responding to cold stress, Gene Ontology (GO) enrichment analysis was performed for TF sets identified at 6 h, 12 h, 24 h, and their intersection (Figure 2). The analyses suggest that transcriptional regulation and RNA-related core biological processes are dominant across all time points; however, the quantitative intensity of these regulatory responses varies between phases. During the early shock phase (6 h), terms such as “regulation of transcription,” “RNA biosynthetic processes,” and “gene expression” are significantly enriched with extremely low FDR values (FDR < 10^−120^). This enrichment suggests that the early-phase response involves the rapid, large-scale mobilization of fundamental transcriptional processes.

The 12 h time point represents the peak phase in terms of the absolute number of differentially expressed TFs. However, the statistical GO enrichment of specific GO terms (e.g., Fold Enrichment and −log10 *p*-values) is quantitatively lower compared to the 6 h and 24 h time points (Figure 2). Despite this relative reduction in enrichment intensity, core processes related to transcription and RNA metabolism remain significantly represented during this intermediate phase.

At the 24 h acclimation phase (24 h), core transcriptional processes exhibit a modest increase in enrichment levels. The preservation of regulatory terms highlighted in the early phase (6 h) suggests that functions associated with transcriptional regulation and RNA biosynthesis are actively maintained during the later stages of cold exposure.

The core TF set consisting of 147 genes shared across the 6 h, 12 h, and 24 h time points exhibits the strongest and most consistent GO enrichment pattern. Within this intersection, terms related to the regulation of transcription, RNA biosynthetic processes, and gene expression stand out with very low FDR values and high gene counts. These 147 shared TFs show consistent enrichment across all time points, suggesting a stable regulatory core.

### 3.3. WGCNA and Significant Modules

According to the WGCNA results, the black, blue, and pink modules exhibit the highest mean kWithin values among all modules (mean kWithin ≈ 4600, 4440, and 3800, respectively). These high intramodular connectivity values indicate dense co-expression relationships within these modules. Based on their strong internal connectivity, the black, blue, and pink modules were defined as the principal regulatory modules of the cold stress response (Supplementary Materials Figure S3). To determine the biological significance of the identified network, module-trait relationships were analyzed by correlating module eigengenes with the distinct cold stress time points (Supplementary Figure S3). This analysis revealed robust temporal associations, demonstrating that specific co-expression modules were dynamically activated in a phase-dependent manner. For instance, the green and blue modules showed strong positive correlations with the early shock phase (6 h), whereas the pink and tan modules were significantly associated with the late acclimation phase (24 h). These strong temporal correlations suggest that these specific phase-associated modules are relevant for downstream regulatory network and hub gene analyses.

#### WGCNA and Topological Hub Genes

**Figure 3:**
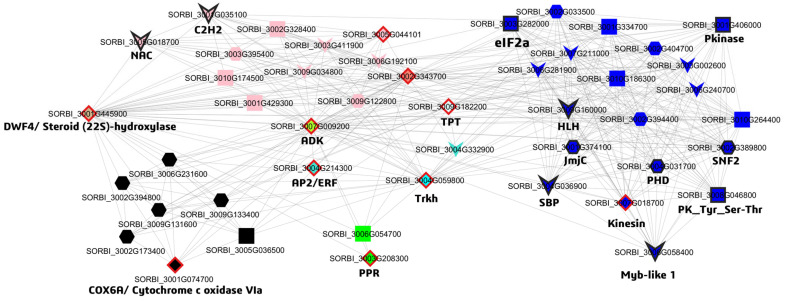
Simplified WGCNA co-expression network under sorghum cold stress. Only high-confidence TOM edges (>0.5) are shown. Nodes represent genes with strong module membership (kME ≥ 0.90) and significant differential expression (padj < 0.01). TFs are shown as inverted triangles, kinases as squares, and other regulators as octagons. Hub genes are shown with red diamond outlines; additional topologically relevant genes are indicated with black borders.

In the WGCNA, modules were identified as groups of co-expressed genes that represent distinct biological processes and temporal phases of the cold response. The structural integrity of these modules is anchored by topological hub genes—network-centric nodes that exhibit the highest intra-modular connectivity (kME) regardless of their specific biochemical function. For instance, the topological hub gene of the black module, *SORBI_3001G074700* (the homolog of *COX6A*), is consistently downregulated at 12 h and 24 h (log_2_FC_12h→24h_ = −1.36 → −1.29, padj_12h→24h_ = 7.51 × 10^−45^ → 3.07 × 10^−44^; kME_black = 0.996) (Figure 3). Another topological hub gene, *SORBI_3007G009200* (annotated as an *adenylate kinase homolog*, *ADK1*), is downregulated only at 24 h (log_2_FC_24h_ = −1.26; padj_24h_ = 1.79 × 10^−32^; kME_greenyellow = 0.982). Additionally, the hub gene *SORBI_3002G343700*, although lacking a clear functional annotation, is also significantly downregulated at 24 h (log_2_FC_24h_ = −1.21; padj_24h_ = 1.36 × 10^−7^; kME_salmon = 0.978). Conversely, the hub gene of the blue module, *SORBI_3007G018700* (*kinesin*), is significantly induced at 6 h and maintains a sustained upregulation at 12 h (log_2_FC_6h→12h_ = 1.6 → 1.66, padj_6h→12h_ = 3.86 × 10^−44^→ 4.74 × 10^−68^; kME_blue = 0.994).

The hub gene of the pink module, *SORBI_3005G044101*, shows a continuously increasing expression profile from 6 h to 24 h (log_2_FC_6h→12h→24h_ = 2.78 → 6.67 → 8.04, padj_6h→12h→24h_ = 1.01 × 10^−3^ → 8.58 × 10^−15^→ 1.04 × 10^−24^; kME_pink = 0.996).

The topological hub gene *SORBI_3001G445900* is annotated as a cytochrome P450 family steroid-related protein. It shows strong repression at 6 h (log_2_FC_6h→24h_ = −1.2 → 1.38, padj_6h→24h_ = 7.4 × 10^−25^ → 1.54 × 10^−22^; kME_tan = 0.982), followed by re-induction at 24 h. In addition, *SORBI_3001G445900* undergoes alternative splicing at 12 h and 24 h. Despite its relatively low intramodular connectivity (mean kWithin ≈ 416), the tan module exhibits high intermodular connectivity (mean kOut ≈ 16,600). Similarly, the cyan and lightcyan modules display low intramodular connectivity (mean kWithin ≈ 348 and 251, respectively) but very high intermodular connectivity (mean kOut ≈ 20,100 and 13,600). Within these highly interconnected modules, the hub gene of the cyan module, *SORBI_3004G059800* (*HKT* homolog), shows activation during the early phase (log_2_FC_6h_ = 1.91; padj_6h_ = 5.09 × 10^−56^; kME_cyan = 0.982), whereas the hub gene of the lightcyan module, *SORBI_3009G182200* (*triose phosphate transporter* homolog, *SbTPT*), is repressed in response to cold shock (log_2_FC_6h_ = −1.05; padj_6h_ = 1.59 × 10^−16^; kME_lightcyan = 0.980).

Within the central network, the AP2-domain-containing *SORBI_3004G214300*, although exhibiting low basal expression, shows a high module membership value (kME_turquoise = 0.980). The *PPR* homolog *SORBI_3003G208300* is identified as the hub gene of the green module and displays downregulation (log_2_FC_6h→12h_ = −3.56 → −4.27, padj_6h→12h_ = 1.77 × 10^−129^ → 1.88 × 10^−158^; kME_green = 0.992). No clear alternative splicing pattern is reported for these genes.

### 3.4. Dynamic Mechanisms of Cold Stress-Induced Transcription Factors

#### 3.4.1. TFs Involved in Shock (6 h) and Early Defense Responses (6 h and 12 h)

**Figure 4:**
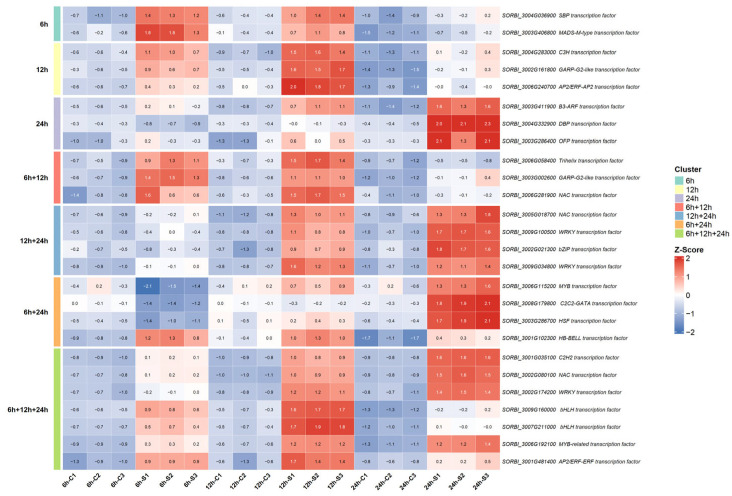
Heatmap illustrating the expression dynamics of differentially expressed transcription factors in sorghum under cold stress at 6 h, 12 h, and 24 h. Rows represent individual transcription factors (Gene ID and family name), grouped according to their temporal response patterns (Cluster color bar on the left). Columns correspond to row-wise Z-score-normalized expression values (C: Control; S: Stress). Note: Color intensity in the heatmap reflects relative expression levels (Red: Up-regulation; blue: Down-regulation), which is independent of the functional WGCNA module names (e.g., blue, pink, tan) mentioned in the text. Only genes with adjusted *p*-value (padj) < 0.01 and strong module association (kME > 0.90) are shown.

To examine the transcriptional responses of sorghum to cold stress, differentially expressed transcription factors (TFs) were visualized on a time-series-based heatmap (Figure 4). This visualization was generated using row-wise Z-score normalization to emphasize relative changes between time points rather than absolute expression levels.

The 6 h time point is characterized by significant transcriptional changes primarily localized within the blue module. In the WGCNA, TFs with high module membership (kME ≥ 0.90) constitute intramodular hub genes of this early phase. Within the blue module, the SBP transcription factor *SORBI_3004G036900* is significantly induced at 6 h (log_2_FC_6h_ = 1.27; padj_6h_ = 4.98 × 10^−29^; kME_blue = 0.97) and exhibits SE-type alternative splicing at 12 h and 24 h. Likewise, the MADS-M-type (Type I) transcription factor *SORBI_3003G406800* is upregulated at 6 h (log_2_FC_6h_ = 1.04; padj_6h_ = 5.73 × 10^−19^; kME_blue = 0.91) and exhibits SE-type splicing between 6 h and 12 h.

During the early defense phase (6 h and 12 h), the Trihelix transcription factor *SORBI_3006G058400* shows strong induction (log_2_FC_6h→12h_ = 1.63 → 1.70; padj_6h→12h_ = 3.92 × 10^−19^ → 4.41 × 10^−25^; kME_blue = 0.97) and exhibits A5SS-type alternative splicing at 12 h, indicating diversification at the post-transcriptional level. Within the same module, the GARP-G2-like factor *SORBI_3003G002600* is upregulated at 6 h and 12 h (log_2_FC_6h→12h_ = 1.32 → 1.03; padj_6h→12h_ = 2.52 × 10^−49^ → 2.06 × 10^−37^; kME_blue = 0.95) and is subjected to MXE- and SE-type splicing between 12 h and 24 h. In this phase, the NAC transcription factor *SORBI_3006G281900*, induced during the early phase (log_2_FC_6h→12h_ = 1.64 → 1.63; padj_6h→12h_ = 1.28 × 10^−9^ → 6.0 × 10^−21^; kME_blue = 0.94), undergoes A5SS-type alternative splicing at 12 h. Within the green module, the C2H2 factor *SORBI_3003G396600* is repressed at 6 h and strongly upregulated at 12 h (log_2_FC_6h→12h_ = −2.33 → 1.43; padj_6h→12h_ = 2.5 × 10^−41^ → 5.88 × 10^−17^; kME_green = 0.91). This gene shows high BLAST similarity to *Arabidopsis HDT3* (E-value = 7.72 × 10^−23^; Bitscore = 541; 44% identity) and is separated from other homologs (*SORBI_3009G260600*, *SORBI_3009G211700*, *SORBI_3010G237800*). In addition, *SORBI_3003G396600* displays strong co-expression within the green module with *SORBI_3006G143200*, which encodes an RNA helicase, and *SORBI_3002G234300*, which encodes a nucleolar protein, with edge weights > 0.99. This co-expression is observed among transcriptional regulators, chromatin-associated factors, and nucleolar proteins (Supplementary Materials Figure S4).

Circadian clock components show high centrality within the blue module. The *CCA1* homolog *SORBI_3004G281800* is upregulated at 6 h and 12 h (log_2_FC_6h→12h_ = 2.15 → 1.83; padj_6h→12h_ = 5.85 × 10^−146^ → 6.73 × 10^−113^; kME_blue = 0.98) and does not display a significant change at 24 h. Another *CCA1* homolog, *SORBI_3010G004300*, is upregulated at 6 h and 12 h (log_2_FC_6h→12h_ = 1.26 → 1.51; padj_6h→12h_ = 1.09 × 10^−51^ → 1.78 × 10^−63^; kME_blue = 0.99) and does not show differential expression at 24 h. Among these two genes, *SORBI_3010G004300* displays a direct connection with edge weight > 0.98 to *SORBI_3003G282000* (*PEK_PEK*, or *SPA3*/*SPA1-RELATED 3*; *SbeIF2α-1*). This specific connection is not detected for *SORBI_3004G281800*. In addition, *SORBI_3010G004300* and *SORBI_3004G281800* are not directly connected to each other within the network (Supplementary Materials Figure S5). Located at the center of the blue module, *SbeIF2α-1* shows high similarity to the *Arabidopsis* SPA3 protein. Within the same module, *SORBI_3001G122832* (*MAPKKK*) is associated with stress signal transduction, while *SORBI_3003G179100* (*Prp40*) is linked to pre-mRNA processing.

#### 3.4.2. TFs in the Acclimation Phase (12 h) and the Transcriptional Peak (12 h and 24 h)

At the 12 h time point, the regulatory network maintains its central structure around the blue module, with high module membership (kME) values for specific hub TFs. In particular, the simultaneous upregulation of the C3H factor (*SORBI_3004G283000*; log_2_FC_12h_ = 1.18; padj_12h_ = 2.59 × 10^−39^; kME_blue = 0.96) and the GARP-G2-like factor (*SORBI_3002G161800*; log_2_FC_12h_ = 1.32; padj_12h_ = 3.85 × 10^−53^; kME_blue = 0.96) is observed. The strong upregulation of the AP2/ERF-AP2 family member *SORBI_3006G240700* (log_2_FC_12h_ = 1.67; padj_12h_ = 2.72 × 10^−37^; kME_blue = 0.90) together with concurrent SE-type alternative splicing is also notable at this time point.

At 12 h and 24 h, the regulatory focus shifts from the blue module to the pink module, associated with the establishment of late-phase regulation. At the center of this restructured network, the NAC factor (*SORBI_3005G018700*; log_2_FC_12h→24h_ = 2.53 → 2.71; padj_12h→24h_ = 2.14 × 10^−37^ → 1.23 × 10^−32^; kME_pink = 0.98), the WRKY factor (*SORBI_3009G100500*; log_2_FC_12h→24h_ = 1.64 → 2.44; padj_12h→24h_ = 1.57 × 10^−52^ → 7.33 × 10^−132^; kME_pink = 0.97), and the bZIP factor (*SORBI_3002G021300*; log_2_FC_12h→24h_ = 4.21 → 5.78; padj_12h→24h_ = 1.52 × 10^−8^ → 5.11 × 10^−16^; kME_pink = 0.95) show near-complete alignment with the pink module eigengene. Specifically, the A5SS-type alternative splicing event observed at 24 h in the WRKY family member *SORBI_3009G034800* (log_2_FC_12h→24h_ = 2.12 → 2.19; padj_12h→24h_ = 1.39 × 10^−66^ → 2.85 × 10^−53^; kME_pink = 0.95) indicates changes in isoform composition.

#### 3.4.3. TFs Associated with the Transition from Early Shock to Late Acclimation (6 h and 24 h)

The expression profiles observed at 6 h and 24 h of cold stress indicate that the early-phase response is reorganized during the acclimation process. This transition is shaped by biphasic regulatory transcription factors that are repressed during the shock phase but strongly upregulated at the late phase. In this context, the MYB transcription factor *SORBI_3006G115200* (log_2_FC_6h→24h_ = −1.94 → 1.79; padj_6h→24h_ = 9.72 × 10^−4^ → 3.65 × 10^−6^; kME_tan = 0.94) and the HSF transcription factor *SORBI_3003G286700* (*SbHSF*; log_2_FC_6h→24h_ = −1.28 → 3.95; padj_6h→24h_ = 9.34 × 10^−6^ → 5.67 × 10^−94^; kME_tan = 0.96)*,* both located in the tan module, shift from repression at 6 h to significant upregulation at 24 h. In particular, the significant increase observed for *SbHSF* is consistent with its functional annotation related to protein folding. Concurrently, C2C2-GATA factor *SORBI_3008G179800*, which represents a central node within the tan module, shows strong upregulation at 24 h following strong repression at 6 h (log_2_FC_6h→24h_ = −3.42 → 4.87; padj_6h→24h_ = 1.38 × 10^−30^ → 2.33 × 10^−184^; kME_tan = 0.97). The high correlation of this gene with the module eigengene indicates strong module association following the initial transcriptional decrease. In contrast to these biphasic expression shifts, the HB-BELL transcription factor *SORBI_3001G102300* in the blue module maintains stable positive upregulation at both 6 h and 24 h (log_2_FC_6h→24h_ = 1.20 → 1.09; padj_6h→24h_ = 1.65 × 10^−35^ → 1.15 × 10^−19^; kME_blue = 0.91).

#### 3.4.4. Acclimation Phase: TF Dynamics in Establishing the New Homeostatic Balance at 24 h

By 24 h, the response shifts to late acclimation with distinct regulatory patterns for each module. At this stage, the pink module component SbB3-ARF (SORBI_3003G411900, log_2_FC_24h_ = 1.55; padj_24h_ = 1.34 × 10^−65^; kME_pink = 0.94) shows induction specifically at 24 h. An MXE-type alternative splicing event was also detected for this gene. Concurrently, SORBI_3004G332900, a DBP factor, located in the tan module (SbDBP; log_2_FC_24h_ = 4.24; padj_24h_ = 6.68 × 10^−196^; kME_tan = 0.91), shows very significant transcriptional upregulation at 24 h. An SE-type alternative splicing event was also detected for SORBI_3004G332900. In contrast, the OFP factor SORBI_3003G286400, also located in the pink module (log_2_FC_24h_ = 4.53; padj_24h_ = 5.56 × 10^−6^; kME_pink = 0.91), is upregulated to a similar extent but does not show any detectable alternative splicing event.

#### 3.4.5. Expression Dynamics of Phase-Independent TFs

**Figure 5:**
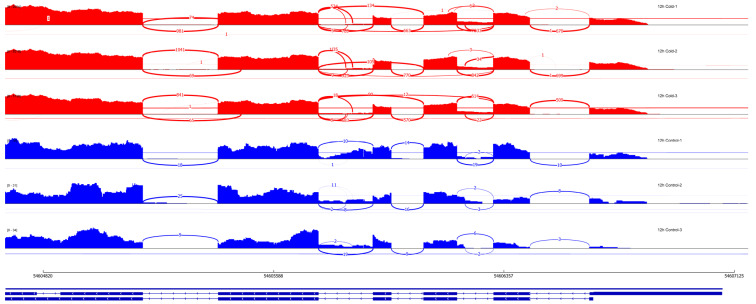
IGV visualization of cold stress-induced alternative splicing in the MYB-related transcription factor *SORBI_3006G192100*. The transcriptomic profile of the locus on Chromosome 6 (Chr06:54,604,437–54,606,745) is shown across six biological replicates. The top three tracks (red) represent samples under 12 h cold stress (Data Range: 0–1,200), while the bottom three tracks (blue) represent control group samples (Data Range: 0–30). Horizontal histograms indicate read coverage depth. Numerical values on the junction arcs (e.g., 7,833 in cold vs. 19 in control) represent the count of RNA-seq reads supporting specific splicing events. A prominent cold-specific intron retention (IR) and alternative splice site pattern is clearly visible, highlighting the stress-responsive isoform regulation of this core pink module component. Gene models at the base are shown according to the NCBI v3.62 annotation.

Transcription factors showing sustained positive regulation across 6 h, 12 h, and 24 h represent a group of genes with continuous expression throughout the shock and acclimation phases. Within the pink module, the central nodes C2H2 (*SORBI_3001G035100*; log_2_FC_6h→12h→24h_ = 4.09 → 6.43 → 9.04; padj_6h→12h→24h_ = 1.92 × 10^−37^ → 1.20 × 10^−101^ → 7.82 × 10^−216^; kME_pink = 0.99) and NAC (*SORBI_3002G080100*; log_2_FC_6h→12h→24h_ = 1.34 → 2.85→ 3.52; padj_6h→12h→24h_ = 1.10 × 10^−49^ → 2.50 × 10^−252^ → 7.85 × 10^−294^; kME_pink = 0.98) show a progressive and significant increase in expression from 6 h to 24 h. Similarly, the WRKY family member *SORBI_3002G174200* (log_2_FC_6h→12h→24h_ = 1.14 → 2.99 → 3.52; padj_6h→12h→24h_ = 2.5 × 10^−9^ → 7.97 × 10^−111^ → 3.67 × 10^−102^; kME_pink = 0.98) *supports* this gradual induction pattern. Another core component of the module, the MYB-related factor *SORBI_3006G192100* (log_2_FC_6h→12h→24h_ = 4.19 → 6.19 → 8.84; padj_6h→12h→24h_ = 2.38 × 10^−117^ → <1.0 × 10^−300^ → 4.1 × 10^−85^; kME_pink = 0.93), is also associated with MXE- and SE-type alternative splicing events across all time points (Figure 5).

In contrast to the pink module, continuity within the blue module is maintained through bHLH and AP2/ERF transcription factors. Among the bHLH factors in the dataset, *SORBI_3009G160000* (log_2_FC_6h→12h→24h_ = 1.55 → 2.47 → 1.37; padj_6h→12h→24h_ = 3.36 × 10^−45^ → 3.53 × 10^−114^ → 5.50 × 10^−20^; kME_blue = 0.98) and *SORBI_3007G211000* (log_2_FC_6h→12h→24h_ = 1.16 → 2.15 → 1.01; padj_6h→12h→24h_ = 1.40 × 10^−34^ → 1.14 × 10^−111^ → 5.13 × 10^−24^; kME_blue = 0.95) maintain a transient yet persistent induction across all three time points. The SE-type alternative splicing observed for *SORBI_3007G211000* at 24 h indicates post-transcriptional regulation during the late phase. In addition, the AP2/ERF-ERF factor *SORBI_3001G481400* (log_2_FC_6h→12h→24h_ = 2.73 → 3.04 → 1.45; padj_6h→12h→24h_ = 3.32 × 10^−93^ → 4.84 × 10^−47^ → 7.81 × 10^−19^; kME_blue = 0.92) maintains its strong induction from 6 h through 24 h.

### 3.5. Signal Transduction Kinases and Transcriptional Regulators Involved in Cold Responses in WGCNA

#### 3.5.1. TRs and PKs in Shock (6 h) and Early Defense Responses (6 h and 12 h)

**Figure 6:**
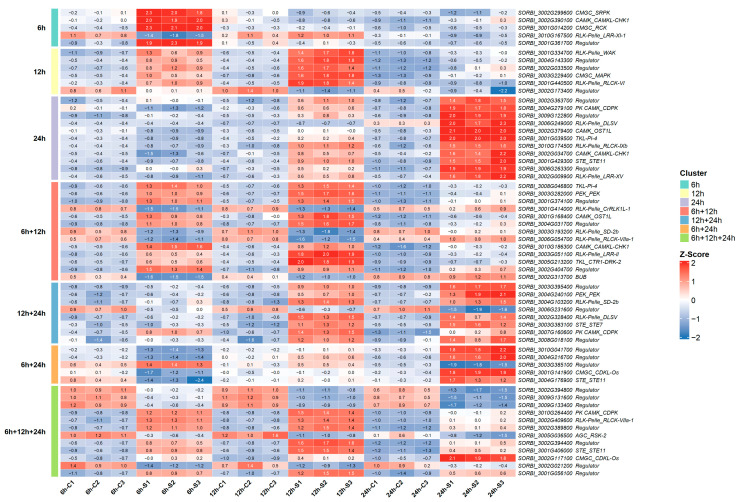
Heatmap showing the expression dynamics of differentially expressed kinases and regulatory proteins in sorghum under cold stress at 6 h, 12 h, and 24 h. Rows represent individual genes (Gene ID and family), grouped by temporal response patterns (left cluster color bar); columns display row-wise Z-score-normalized expression values (C: Control; S: Stress). Color intensity indicates relative expression (red: up-regulation; blue: down-regulation) and is independent of WGCNA module names (e.g., blue, pink, tan). Only genes with padj < 0.01 and strong module membership (kME > 0.90) are included.

The differential expression of PKs and TRs involved in the cold stress response in sorghum was visualized across three time points using a heatmap (Figure 6) and detailed in Supplementary Materials Table S4. Row-wise Z-score normalization was applied to emphasize temporal changes, enabling the identification of specific windows of upregulation or repression for each gene. Temporal patterns were interpreted based on Z-score values, while the magnitude of expression changes was evaluated using the corresponding log_2_FC values.

At the 6 h time point, kinase activation was primarily detected in the cyan module. The upregulation of *CMGC_SRPK-1* (*SORBI_3002G299600*; log_2_FC_6h_ = 1.04; padj_6h_ = 1.69 × 10^−13^; kME_cyan = 0.94) and *CMGC_RCK* (*SORBI_3001G014200*; log_2_FC_6h_ = 1.02; padj_6h_ = 2.69 × 10^−37^; kME_cyan = 0.92) was observed at this stage. *CAMK_CAMKL-CHK1-1* (*SORBI_3002G390100*; log_2_FC_6h_ = 1.22; padj_6h_ = 8.41 × 10^−57^; kME_cyan = 0.93), a kinase associated with cell cycle control, was also upregulated. The transcriptional regulator *SbIWS1-1* (*SORBI_3001G361700*; log_2_FC_6h_ = 2.08; padj_6h_ = 3.58 × 10^−57^; kME_cyan = 0.91) was also upregulated at 6 h. In contrast, *SORBI_3010G167500* (*SbRLK-Pelle_LRR-XI-1*; log_2_FC_6h_ = −1.51; padj_6h_ = 2.52 × 10^−47^; kME_lightcyan = 0.92) was repressed in the lightcyan module.

As the response progresses toward the 12 h time point, translational and epigenetic regulatory components become more prominent within the blue module. The *TKL-Pl-4-1* kinase (*SORBI_3008G046800;* log_2_FC_6h→12h_ = 1.32 → 1.33; padj_6h→12h_ = 6.89 × 10^−37^ → 8.14 × 10^−57^; kME_blue = 0.99) shows stable upregulation across this interval. At the same time, increased expression of the protein synthesis-associated factor *SbeIF2α-1* (*SORBI_3003G282000*, log_2_FC_6h→12h_ = 1.82 → 2.59; padj_6h→12h_ = 9.13 × 10^−103^ → 5.17 × 10^−227^; kME_blue = 0.99) and the ABA-related *CAMK_OST1L-1* kinase (*SORBI_3001G168400*; log_2_FC_6h→12h_ = 1.26 → 1.56; padj_6h→12h_ = 1.77 × 10^−21^ → 7.23 × 10^−21^; kME_blue = 0.98) suggests the activation of translational and epigenetic regulatory components. In addition, an SE-type alternative splicing event is detected for SbeIF2α-1 at 24 h.

An increase in the expression of receptor-like kinases is also observed in the blue module, including *RLK-Pelle_LRR-II* (*SORBI_3003G051100*, log_2_FC_6h→12h_ = 1.50 → 2.96; padj_6h→12h_ = 6.41 × 10^−29^ → 2.76 × 10^−138^; kME_blue = 0.95) and *TKL_CTR1-DRK-2* (*SORBI_3005G213200*; log_2_FC_6h→12h_ = 2.09 → 4; padj_6h→12h_ = 2.18 × 10^−27^ → 2.25 × 10^−154^; kME_blue = 0.94).

The upregulation of chromatin remodelers, including *SORBI_3001G374100* (*SbJumonji*; log_2_FC_6h→12h_ = 1.60→ 1.52; padj_6h→12h_ = 6.29 × 10^−54^ → 2.85 × 10^−57^; kME_blue = 0.98), *SbSNF2-1* (*SORBI_3002G404700*; log_2_FC_6h→12h_ = 1.27 → 1.09; padj_6h→12h_ = 1.28 × 10^−52^ → 2.93 × 10^−30^; kME_blue = 0.92), and *PHD* (*SORBI_3004G031700*; log_2_FC_6h→12h_ = 1.51 → 1.96; padj_6h→12h_ = 6.46 × 10^−53^ → 1.32 × 10^−98^; kME_blue = 0.97) is observed at this stage. In contrast, the green module is characterized by the significant downregulation of receptor kinases, including *RLK-Pelle_CrRLK1L-1* (*SORBI_3001G414000*; log_2_FC_6h→12h_ = −1.67→ −1.63; padj_6h→12h_ = 4.07 × 10^−47^ → 9.87 × 10^−58^; kME_green = 0.98) and *RLK-Pelle_SD-2b-1* (*SORBI_3003G193200*; log_2_FC_6h→12h_ = −1.30 → −1.67; padj_6h→12h_ = 3.66 × 10^−26^ → 1.87 × 10^−35^; kME_green = 0.97), together with the cytoplasmic *RLK-Pelle_RLCK-VIIa-1* (*SORBI_3006G054700*; log_2_FC_6h→12h_ = −2.97 → −3.65; padj_6h→12h_ = 5.36 × 10^−46^ → 3.92 × 10^−63^; kME_green = 0.95) and the mitotic kinase *BUB* (*SORBI_3002G313700*; log_2_FC_6h→12h_ = −1.64 → −1.12; padj_6h→12h_ = 9.13 × 10^−24^ → 4.02 × 10^−12^; kME_green = 0.92). The significant downregulation of *RLK-Pelle_RLCK-VIIa-1* is also accompanied by an RI-type alternative splicing event.

#### 3.5.2. TRs and PKs in the Acclimation Phase (12 h) and the Transcriptional Peak (12 h and 24 h)

At 12 h, the blue module represents a selective activation pattern distinct from the early shock phase. At this stage, cell surface-associated receptors, cytoplasmic kinases, and nuclear regulatory proteins show coordinated involvement. In this regard, the blue module at 12 h is characterized by cell wall-associated signaling components and nuclear-level regulatory reprogramming. Cell wall-associated receptor kinase *RLK-Pelle_WAK (SORBI_3001G334700,* log_2_FC_12h_ = 1.16; padj_12h_ = 9.01 × 10^−25^; kME_blue = 0.98) is upregulated at 12 h following an RI-type alternative splicing (AS) event detected at 6 h. Concurrently, the transcriptional regulators *ARID* (*SORBI_3004G143300*; log_2_FC_12h_ = 1.36; padj_12h_ = 2.07 × 10^−58^; kME_blue = 0.97) and *Rcd1-like* (*SORBI_3002G033500*; log_2_FC_12h_ = 1.02; padj_12h_ = 2.01 × 10^−12^; kME_blue = 0.97) are upregulated at 12 h. The *Rcd1-like* gene additionally exhibits SE-type alternative splicing at 24 h. In cytosolic signaling, *CMGC_MAPK* (*SORBI_3003G229400*; log_2_FC_12h_ = 1.2; padj_12h_ = 1.49 × 10^−42^; kME_blue = 0.95) and RLK-Pelle_RLCK-VI (*SORBI_3001G440500*; log_2_FC_12h_ = 1.69; padj_12h_ = 2.46 × 10^−47^; kME_blue = 0.93) are upregulated at 12 h. SANTA domain-containing protein (*SbSANTA1*) in the black module (*SORBI_3002G173400*; log_2_FC_12h_ = −1.32; padj_12h_ = 2.06 × 10^−7^; kME_black = 0.91) is downregulated and exhibits A5SS-type alternative splicing. At the 12 h → 24 h transition, increased expression is observed in the pink module. In this phase, *SORBI_3003G395400* (*SbTAZ-1*), a transcriptional regulator, shows a progressive increase in expression (log_2_FC_12h→24h_ = 3.20 → 5.09; padj_12h→24h_ = 1.50 × 10^−108^ → <1.0 × 10^−300^; kME_pink = 0.98). SE-type alternative splicing events at 6 h and 24 h, together with an MXE-type event at 24 h, are detected for this gene. A protein synthesis-associated kinase component also shows a progressive increase during the late phase, represented by *SORBI_3004G240100* (*PEK_PEK*, *SbeIF2α-2*) (log_2_FC_12h→24h_ = 3.03 → 5.12; padj_12h→24h_ = 1.34 × 10^−4^ → 1.21 × 10^−9^; kME_pink = 0.95). Within the receptor kinase signaling pathway, *SORBI_3004G103200* (*SbRLK-Pelle_SD-2b-2*; log_2_FC_12h→24h_ = 4.92 → 5.47; padj_12h→24h_ = 9.24 × 10^−11^ → 3.19 × 10^−9^; kME_pink = 0.95) and *SORBI_3002G328400* (*SbRLK-Pelle_DLSV-1*; log_2_FC_12h→24h_ = 1.83 → 1.65; padj_12h→24h_ = 1.77 × 10^−39^ → 7.46 × 10^−19^; kME_pink = 0.93) display sustained induction. An SE-type alternative splicing event was observed for *SORBI_3002G328400*. In addition, another component of the MAPK pathway, *SORBI_3003G383100* (*SbSTE_STE7*; log_2_FC_12h→24h_ = 4.13 → 5.07; padj_12h→24h_ = 5.65 × 10^−7^ → 9.68 × 10^−8^; kME_pink = 0.93), reaches its highest expression level at 24 h. The calcium-dependent kinase *SORBI_3007G160600* (*SbCAMK_CDPK-1*; log_2_FC_12h→24h_ = 1.41 → 1.10; padj_12h→24h_ = 9.29 × 10^−32^ → 1.04 × 10^−10^; kME_blue = 0.93) maintains positive expression across this interval. At the epigenetic level, the regulator *SORBI_3008G018100* (*SbSET-1*; log_2_FC_12h→24h_ = 3.12 → 3.87; padj_12h→24h_ = 4.88 × 10^−6^ → 1.66 ×10^−6^; kME_pink = 0.91) shows increased expression at 24 h, whereas *SORBI_3006G231600* (*SbGNAT*; log_2_FC_12h→24h_ = −1 → −2.18; padj_12h→24h_ = 3.85 × 10^−9^ → 4.29 ×10^−28^; kME_black = 0.94) remains repressed over the same period.

#### 3.5.3. TRs and PKs Associated with the Transition from Early Shock to Acclimation (6 h and 24 h)

The dynamics of the sorghum transcriptome between 6 h and 24 h indicate a redistribution of regulatory activity across modules, accompanied by reorganization of transcriptional profiles from the early shock response toward late acclimation. This transition is particularly evident within the tan module, where several central genes reverse their expression direction from negative at 6 h to positive at 24 h. Within the tan module, a B-box-type regulator (*SbBbox*), which shows strong correlation with the module eigengene, shifts from repression at 6 h to significant upregulation at 24 h (*SORBI_3010G041700;* log_2_FC_6h→24h_ = −1.35 → 2.66; padj_6h→24h_ = 7.81 × 10^−36^ → 6.26 × 10^−80^; kME_tan = 0.98). Likewise, a comparable directional shift is observed for *SORBI_3004G216700*, a homolog of the circadian clock component *TOC1* (*Pseudo ARR-B*) (log_2_FC_6h→24h_ = −1.86 → 4.01; padj_6h→24h_ = 1.03 × 10^−30^ → 1.7 × 10^−113^; kME_tan = 0.98). The involvement of this gene in an MXE-type alternative splicing event at 24 h indicates that circadian regulation during the late phase is also modulated at the isoform level. The shift in transcriptional focus is further reflected in the expression profile of the *SbTAZ-2* regulator *SORBI_3003G385100* within the greenyellow module. This gene is upregulated at 6 h but significantly downregulated at 24 h (log_2_FC_6h→24h_ = 1.19 → −1.97; padj_6h→24h_ = 1.05 × 10^−27^ → 6.11 × 10^−19^; kME_greenyellow = 0.97). In contrast to its early increase, the reduced expression at 24 h indicates a reversal in transcriptional direction. This opposing pattern is consistent with a temporal shift from early stress-associated expression toward a distinct late-phase regulatory configuration during acclimation. At the level of signal transduction, the kinase *SORBI_3001G141900* (*CMGC_CDKL-Os*) within the tan module shows a significant increase at 24 h (log_2_FC_6h→24h_ = −2.71 → 3.77; padj_6h→24h_ = 6.98 × 10^−4^ → 4.68 × 10^−15^; kME_tan = 0.97), shifting from reduced expression at 6 h to higher expression at 24 h. In addition, *SORBI_3004G176900* (*SbSTE_STE11-1*), positioned upstream in the MAPK cascade, displays increased transcript levels and is associated with MXE- and SE-type alternative splicing events at 12 h and 24 h (log_2_FC_6h→24h_ = −1.36 → 1.0; padj_6h→24h_ = 3.23 × 10^−13^ → 1.82 × 10^−13^; kME_tan = 0.91), indicating concurrent transcriptional and isoform-level changes during this transition.

#### 3.5.4. Regulatory Responses in the Acclimation Phase: TR and PK Dynamics at 24 h

The 24 h time point (late acclimation) represents a phase distinct from the early shock stage, characterized by activation of both cytosolic kinase cascades and nuclear transcriptional regulators. At this stage, regulatory activity increases in the pink and tan modules, where signaling and transcriptional control components show increased expression. Within the tan module, genes with high intramodular connectivity (kME_tan ≥ 0.91) are associated with calcium-mediated signaling and stomatal regulation during late acclimation. In this context, the calcium-dependent protein kinase *SbCAMK_CDPK-2* (*SORBI_3004G279100*; log_2_FC_24h_ = 1.53; padj_24h_ = 1.02 × 10^−73^; kME_tan = 0.94) and the stomatal regulation-associated kinase *SORBI_3002G379400* (*SbCAMK_OST1L-2*; log_2_FC_24h_ = 2.17; padj_24h_ = 2.75 × 10^−78^; kME_tan = 0.92) show strong induction at 24 h. An MXE-type alternative splicing event was detected at 12 h in *CAMK_OST1L-2.* In addition, within the same module, the upregulation of *SORBI_3001G039500* (*TKL-Pl-4-2*; log_2_FC_24h_ = 2.36; padj_24h_ = 2.21 × 10^−203^; kME_tan = 0.92), *SORBI_3002G034700* (*CAMK_CAMKL-CHK1-2;* log_2_FC_24h_ = 1.15; padj_24h_ = 1.37 × 10^−18^; kME_tan = 0.92) and *SORBI_3002G009900* (*RLK-Pelle_LRR-XV;* log_2_FC_24h_ = 1.72; padj_24h_ = 3.67 × 10^−27^; kME_tan = 0.91) are upregulated at 24 h. SE-type AS events are detected in TKL-Pl-4-2 and CAMKL-CHK1-2 at this stage.

The pink module is associated with chromatin remodeling and epigenetic modification processes. Genes with high module membership (kME_pink ≥ 0.91), including *SbSNF2-2* (*SORBI_3002G363700*, log_2_FC_24h_ = 1.41; padj_24h_ = 8.74 × 10^−10^; kME_pink = 0.96) and the *SbSET-2* regulator *(SORBI_3009G122800*; log_2_FC_24h_ = 1.51; padj_24h_ = 2.06 × 10^−10^; kME_pink = 0.93), show increased expression at 24 h. An SE-type alternative splicing event was detected for SbSET-2 at 12 h. In the same module, the upstream MAPK cascade component *SORBI_3001G429300* (*SbSTE_STE11-2*; log_2_FC_24h_ = 1.74; padj_24h_ = 1.47 × 10^−44^; kME_pink = 0.91) and the response regulator (*SbRR*) (*SORBI_3006G263300*; log_2_FC_24h_ = 2.10; padj_24h_ = 1.30 × 10^−51^; kME_pink = 0.91) show clear induction at 24 h. SE- and MXE-type alternative splicing events are detected for *SORBI_3006G263300*. Cell surface sensing mechanisms are reflected in the turquoise module by the strong induction of the central receptor kinase *SbRLK-Pelle_DLSV-2* (*SORBI_3006G249000*; log_2_FC_24h_ = 3.34; padj_24h_ = 2.25 × 10^−10^; kME_turquoise = 0.92).

#### 3.5.5. Expression Dynamics of Phase-Independent TRs and PKs

Regulators that exhibit consistent expression patterns across all time points (6 h, 12 h, and 24 h) were identified across modules. WGCNA identified two black intra-module hubs, *SORBI_3002G394800* (*SWI/SNF-BAF60b;* log_2_FC_6h→12h→24h_ = −1.2 → −2.15 → −2.46; padj_6h→12h→24h_ = 7.05 × 10^−22^ → 6.77 × 10^−48^ → 9.01 × 10^−45^; kME_black = 0.99) and *SORBI_3005G036500* (*AGC_RSK-2;* log_2_FC_6h→12h→24h_ = −1.94 → −2.91 → −2.56; padj_6h→12h→24h_ = 3.26 × 10^−8^ → 6.10 × 10^−13^ → 1.07 × 10^−4^; kME_black = 0.95), both strongly repressed throughout the time course. The mTERF family members *SORBI_3009G131600* (*mTERF-1*; log_2_FC_6h→12h→24h_ = −1.3 → −2.2 → −2.3; padj_6h→12h→24h_ = 5.08 × 10^−13^ → 6.59 × 10^−34^ → 3.75 × 10^−24^; kME_black = 0.99) and *SORBI_3009G133400* (*mTERF-2*; log_2_FC_6h→12h→24h_ = −1.71 → −2.17 → −2.79; padj_6h→12h→24h_ = 1.84 × 10^−22^ → 1.42 × 10^−33^ → 1.81 × 10^−32^; kME_black = 0.99) are significantly downregulated across all phases.

In the blue module, the calcium-dependent signaling component *SORBI_3010G264400* (*SbCAMK_CDPK-3*) (log_2_FC_6h→12h→24h_ = 1.97 → 2.21 → 1.00; padj_6h→12h→24h_ = 2.96 × 10^−145^ → 1.2 × 10^−170^ → 1.08 × 10^−22^; kME_blue = 0.98) was consistently upregulated across all time points. This gene also exhibits an SE-type alternative splicing event at 24 h. Within the same module, *SORBI_3002G409600* (*RLK-Pelle*) (log_2_FC_6h→12h→24h_ = 2.10 → 2.18 → 1.15; padj_6h→12h→24h_ = 2.17 × 10^−63^ → 1.19 × 10^−109^ → 6.85 × 10^−27^; kME_blue = 0.97) and *SORBI_3001G406000* (*STE_STE11-3*) maintain high induction levels throughout the entire time course (log_2_FC_6h→12h→24h_ = 3.4 → 4.52 → 3.42; padj_6h→12h→24h_ = 3.4 × 10^−122^ → 3.9 × 10^−199^ → 1.1 × 10^−72^; kME_blue = 0.94).

In addition, *SORBI_3001G056100* (*AUX/IAA*), also located in the blue module, exhibits a strong and sustained activation profile across all time points (log_2_FC_6h→12h→24h_ = 3.23 → 3.98 → 3.42; padj_6h→12h→24h_ = 8.2 × 10^−60^ → 8.65 × 10^−87^ → 2.91 × 10^−45^; kME_blue = 0.91). At the level of chromatin modification, regulatory involvement is reflected by the MXE-type alternative splicing event detected at 12 h in *SORBI_3002G389800* (*SbSNF2-3*) (log_2_FC_6h→12h→24h_ = 1.39 → 1.58 → 1.12; padj_6h→12h→24h_ = 1.36 × 10^−48^ → 1.12 × 10^−52^ → 4.83 × 10^−25^; kME_blue = 0.96). Conversely, *SORBI_3002G394400* (*SbIWS1-2*) is induced at all time points and consistently exhibits SE-type alternative splicing (log_2_FC_6h→12h→24h_ = 1.38 → 2.48 → 1.56; padj_6h→12h→24h_ = 1.93 × 10^−40^ → 9.8 × 10^−186^ → 2.4 × 10^−33^; kME_blue = 0.95). In the pink module, *SORBI_3002G117100* (*CMGC_CDKL-Os*) displays progressively increasing induction toward 24 h (log_2_FC_6h→12h→24h_ = 2.17 → 3.17 → 6.17; padj_6h→12h→24h_ = 2.37 × 10^−9^ → 7.65 × 10^−17^ → 8.5 × 10^−35^; kME_pink = 0.92). In contrast, persistent repression of genes such as *SORBI_3002G021200* (*SNF2*) in the green module confirms that specific chromatin regulatory branches remain downregulated throughout the stress period (log_2_FC_6h→12h→24h_ = −3.71 → −3.17 → −1.62; padj_6h→12h→24h_ = 1.27 × 10^−25^ → 2.04 × 10^−14^ → 9.5 × 10^−6^; kME_green = 0.91).

## 4. Discussion

### 4.1. Rapid Ionic Signaling and Metabolic Shutdown

The temporal dynamics of the transcriptomic response of sorghum to cold stress support a working model in which the plant’s initial reaction involves a coordinated, systemic reprogramming rather than a strictly localized response. The repression observed in the energy metabolism genes *COX6A* and *ADK1* suggests that ATP consumption may be actively restricted, potentially adjusting the balance between growth and defense through energy economy. Consistent with previous studies, global protein synthesis is suppressed under cold stress to reduce energy expenditure [45]. As part of this general energy-conservation strategy, repression of *COX6A*, a core component of the mitochondrial respiratory chain, has been reported under cold stress conditions [46]. Similarly, in pepper (*Capsicum annuum*), the repression of *CaADK1*, which begins at 3 h of cold exposure and becomes more pronounced at 12 h and 24 h, has been interpreted as part of a fundamental metabolic response that limits energy consumption. The association of this negative regulation with ABA (6 h), MeJA (24 h), and SA (24 h) signaling further supports the view that an energy economy-based adaptive process operates between 3 h and 24 h of cold stress [47]. In this context, the repression of *COX6A* and *ADK1* observed at 6 h may be associated with a dual energy–redox control strategy by potentially both reducing the translational burden and limiting mitochondrial ROS production. Under cold conditions, the reduction in reactive oxygen species (ROS) that may arise from a damaged respiratory chain has been reported to contribute to the maintenance of cellular integrity [48]. Taken together, these observations suggest that early-phase energy restriction and redox regulation operate in parallel as a protective mechanism.

At the organelle level, PPR proteins have attracted attention. While the upregulation of PPR genes under cold stress is documented [49], specific roles in chloroplast biogenesis and superoxide modulation have been demonstrated in other species [50]. In addition, a mitochondrial PPR protein has been reported to enhance cold tolerance by modulating superoxide levels [51]. In the present study, the early-phase repression of the green module hub *PPR* gene *SORBI_3003G208300* suggests a potential temporary reduction in organelle transcript processing and translational capacity. This pattern is consistent with a limitation of growth-associated mitochondrial and chloroplast activity.

Similarly, repression of the homolog of the lightcyan module topological hub gene *SbTPT* (*SORBI_3009G182200*) suggests a potential restriction in triose-phosphate export from the chloroplast to the cytosol. *SbTPT* has been described as a regulator of starch metabolism through its role in transporting photosynthetic products [52]. In rice, members of the *TPT* family have been reported to exhibit differential expression under cold stress [50]. In the present dataset, this repression may reflect a metabolic adjustment that favors protective sugar retention within the chloroplast and is consistent with a growth–defense tradeoff strategy [53].

Another notable finding is the transcriptional downregulation of receptor kinase associated with the LRR-RLK family during the early phase. The repression of the topological hub *SbRLK-Pelle_LRR-XI-1* identified in this study suggests a potential controlled reduction in signal amplification at the cell surface. These receptor-like cytoplasmic kinase (RLCK) proteins function as a link between calcium/calmodulin (CaM) signaling and the cold response pathway [54,55]. Upon cold perception, they become activated and regulate MAPK cascades, particularly the MEKK1–MKK2–MPK4 pathway, thereby positively contributing to freezing tolerance [54]. In contrast, LRR-RLK members such as *CTB4a* [55], *OsSERL2* [56], *BRI1* [57], and *BAK1* have been described [58] as key initiating receptors involved in sensing cold or cold-induced brassinosteroid signaling. Accordingly, repression of *SbRLK-Pelle_LRR-XI-1* may indicate that, during the early phase, the regulatory focus shifts toward intracellular processing of existing stress signals rather than to the amplification of new signal production.

Potential reorganization of cellular architecture represents an additional component of the early stress response. Kinesin motor proteins function in microtubule organization and have been reported to be activated during the early phase of cold stress due to the direct effects of low temperature on the cytoskeleton. In soybean (*Glycine max*), rapid activation of genes encoding kinesin-like proteins within the first 2 h has been demonstrated in cold-tolerant cultivars [59]. In the present study, the topological hub kinesin gene *SORBI_3007G018700* was markedly induced at 6 h and 12 h. This expression pattern suggests that an adaptive structural reorganization of the cell may occur during the early and peak phases.

### 4.2. The AP2/ERF–NAC Axis and Transcriptional Control of the Cold Response

At the core of the sorghum cold stress response lies a set of 147 genes that remain active across all time points, independent of stress duration, here defined as the “Core Cold Gene Set.” Within this group, members of the AP2/ERF and NAC families stand out as major components contributing to transcriptional regulation of the response [6,9]. In the present study, the identification of SORBI_3005G131900—a bHLH transcription factor showing high orthology to Arabidopsis ICE1 (58% identity, E-value: 5.54 × 10^−88^)—suggests a potential regulatory role within this core circuit (Supplementary Materials Table S5). Although ICE1 is traditionally regulated at the protein level, its sustained transcriptional induction in this dataset suggests a possible continuous de novo synthesis strategy to maintain its pool under stress conditions. As a primary activator of the CBF-dependent pathway, SbICE1 may orchestrate the “Core Cold Gene Set,” a hypothesis that aligns with the observed sustained expression of upstream STE-mediated kinases, although functional validation is required to confirm such post-translational interactions. NAC transcription factors have been described as forming a multilayered upstream regulatory mechanism that reinforces the *CBF/DREB* (*AP2/ERF*) genes, which constitute a central axis of cold stress regulation, at both the transcriptional and protein levels [51,56]. Certain NAC members directly bind to *CBF* promoters, enhancing the expression of genes such as *CBF1* and *CBF3*, while simultaneously activating secondary metabolism pathways (e.g., anthocyanin biosynthesis), thereby contributing to cold tolerance [5,51]. At the level of major transcriptional switches, the AP2/ERF family plays a central role. *SbERF027* (*SORBI_3004G283300*) [9] and *SbCBF6* (*SbEREB35*; *SORBI_3002G269400*/*Sobic.002G269400*) have been reported to be induced in cold-tolerant cultivars [60]. In the present study, both *SbERF027* and *SbCBF6* genes exhibited sustained high expression across all time points (Supplementary Materials Table S5). Although expressed at relatively low levels, the AP2-domain-containing gene *SORBI_3004G214300* in the present study may exert an amplifying effect on downstream regulatory networks, with the potential to influence the expression of numerous target genes. Furthermore, the sustained expression of *SbB3-ARF* in this study suggests that auxin-mediated developmental adjustments might be integrated into this core transcriptional circuit [61,62]. These findings indicate that the CBF/ERF regulatory circuit remains functionally engaged at the transcript level throughout the cold response.

Interestingly, while the 12 h time point represents the peak of transcriptional activity in terms of the absolute number of differentially expressed transcription factors, a relative reduction in the quantitative intensity of GO enrichment is observed during this phase. It is hypothesized that this pattern may reflect a broader biological diversification of regulatory networks. As the plant transitions from the initial shock response (6 h) to early acclimation, transcriptional activity might be distributed across a wider array of secondary metabolic and developmental pathways, thereby potentially diluting the statistical enrichment of core RNA-related terms. Alternatively, this observation could be partially attributed to the inherent limitations of current homology-based functional annotations, as many intermediate-phase regulators in sorghum remain uncharacterized.

### 4.3. Epigenetic Priming

Stabilization of the cold response at the chromatin level and potential regulation of protein synthesis become more prominent during this phase. At 12 h, the module-specific hub kinase *SbeIF2α-1* (generically annotated as SPA3-like in Ensembl, but classified as an eIF2α-like kinase via the plant-specific iTAK pipeline) is upregulated and its expression profile may contribute to a potential controlled restriction of protein synthesis. Such a mechanism is often associated with cold-induced ROS or shock signals that activate the GCN2 kinase, leading to eIF2 phosphorylation and a subsequent reduction in global translation [45]. Upon phosphorylation of *eIF2*, global protein synthesis is reported to be suppressed [63]. This potential translational repression is hypothesized to limit energy-intensive growth processes and facilitate allocation of available energy toward the synthesis of protective stress-related proteins, including those whose expression is consistent with epigenetic regulation mechanisms involving chromatin remodelers such as *SNF2* and *PHD* domain proteins [48,63]. In this study, multiple SNF2 paralogs were detected across different temporal modules. This induction of surface sensors, coupled with the coordinated upregulation of blue module hubs such as the JmjC domain-containing SbJumonji, the ATP-dependent remodeler *SbSNF2-1*, and a PHD finger protein, indicates that chromatin restructuring may progress in tandem with renewed environmental sensing during the 6 h to 12 h transition. These expression patterns suggest that chromatin-related regulation, particularly through SNF2 family members (e.g., *BRM*, *PKL*) which regulate the accessibility of cold-responsive genes, is active from the early phase and continues into stabilization [64].

At 24 h, the pink module was characterized by peak expression of *SbSNF2-2* and *SbSET-2*. Previous studies have reported that histone marks such as H3K4me3 and H3K27me3 are associated with stable chromatin states during long-term acclimation [65,66]. PHD finger proteins act as readers of these marks and physically interact with SNF2 complexes—such as the FORGETTER1 (FGT1)–BRM or CHR11/17 complexes—to regulate nucleosome dynamics and support epigenetic priming during successive temperature fluctuations [64]. In the present study, the elevation of *SbeIF2α-1* to intra-module hub status at 12 h may reflect an energy reallocation strategy that accompanies these proposed chromatin-based memory mechanisms. Fine-tuning of this process likely involves JmjC domain-containing histone demethylases like Jumonji, which are recognized as critical components of epigenetic memory pathways [64]. A potential direct link between Jumonji genes and alternative splicing has been reported. In *Medicago truncatula*, the JmjC domain gene *MtJMJC5* undergoes cold-responsive alternative splicing, producing distinct mRNA isoforms [67].

In contrast to the ABA-centered reactive strategy reported in maize [13,68], the sorghum response appears to be coordinated through a time-gated mechanism synchronized with the circadian clock and involving regulators such as *SbHDT3*, *PIF7*, and *CCA1* [10,69]. The involvement of *SbCAMK_OST1L-2* at this phase is consistent with a model of sustained cold response, as OST1 is known to phosphorylate and stabilize ICE1, thereby maintaining the CBF regulon by preventing proteasomal degradation [55]. As an extension of this circadian regulation, the differential behavior of the two *CCA1* genes in the sorghum genome may suggest functional divergence following genome duplication, where one paralog primarily maintains temporal regulation while the other may contribute to transcriptional signals associated with translational restraint [70]. In addition, the role of *SORBI_3001G300201*, the sorghum ortholog of *PIF7* (Supplementary Materials Table S5), during the acclimation phase is consistent with reports that alternative splicing-mediated intron retention can attenuate *PIF7* function, thereby relieving repression on CBF genes and promoting allocation of energy from growth toward defense [71,72].

*SbHDT3*, a circadian-associated regulator in sorghum, has been characterized as a histone deacetylase involved in chromatin-mediated transcriptional regulation. Its sustained expression pattern in this dataset is consistent with a potential epigenetic contribution to cold adaptation. In addition, an MXE-type alternative splicing event was detected in the chromatin remodeling gene *SbSNF2-3*, which remained highly expressed through 24 h. This gene has previously been reported as a target of miR166 [60]. Together with earlier reports indicating multilayered regulation of cold responses [73], these observations suggest that chromatin-associated regulators may be subject to coordinated transcriptional and post-transcriptional modulation during cold stress.

### 4.4. Regulatory Inversion: The Tan Module and P450 Rebound

The transition from the shock phase to the acclimation phase is associated with a distinct repression–reinduction pattern within the tan module. While the strict topological hub of this module is an uncharacterized steroid-related P450 locus (*SORBI_3001G445900),* this biphasic dynamic is functionally exemplified by the parallel behavior of *SbCYP79A2* (*SORBI_3010G172200*). Although *SbCYP79A2* represents an established cold-responsive literature marker rather than a strict network hub (Supplementary Materials Table S5), its profile provides a mechanistic context for this rebound. While *CYP* genes are typically reported as repressed under cold stress [45], *SbCYP79A2* exhibited a mid-phase transient induction at 12 h in the present dataset, followed by sustained levels at 24 h. In sensitive cultivars, excessive downregulation of *CYP* genes has been associated with membrane instability and oxidative stress [45]. The rebound observed here may therefore reflect a successful phase transition and could indicate a potential protective adjustment involving secondary metabolism and ROS balance as the plant enters the acclimation stage.

Within this biphasic transition, regulatory genes in the tan module—*SbHSF*, *SbBox*, and *MYB*—exhibited a directional shift, being repressed at 6 h and significantly induced at 24 h. This pattern suggests the potential initiation of longer-term acclimation processes. However, a notable exception was observed for the specific B-box gene locus *SORBI_3004G211200* (*Sobic.004G211200*). While B-box proteins are generally recognized as integrators of light and temperature signaling linked to frost tolerance, this gene was significantly downregulated at 24 h (log_2_FC = −1.2; padj = 2.03 × 10^−11^) (Supplementary Materials Table S5).

In contrast to the general induction trend of the tan module, the targeted repression of this B-box member may represent a specialized regulatory checkpoint. This shift is potentially mediated by *SbTAZ-1*, a transcriptional regulator that exhibited a progressive induction in the pink module. In plants, BTB-TAZ proteins—such as MdBT2 in apple [74] or *BT1* in *Arabidopsis*—are known to target cold-tolerance transcription factors for degradation via the ubiquitin-proteasome pathway to balance the growth-defense tradeoff [9]. The robust upregulation of *SbTAZ-1*, coupled with its isoform-level modifications via SE and MXE-type alternative splicing, suggests a possible specialized regulatory role in fine-tuning late-phase acclimation. Furthermore, the contrasting expression pattern of SbTAZ-2 suggests a complex, phase-specific coordination within the TAZ family, potentially acting in concert to modulate the stability of downstream factors such as *B-box* and *TOC1*.

Transcriptomic (RNA-seq) analyses have shown that MYB transcription factors are broadly responsive to cold stress in sorghum [9]. In the cold-tolerant cultivar “*Hongke4*,” *MYB62* (*Sb04g026210*) has been reported to be strongly induced under cold conditions [12]. In this study, the corresponding ortholog *SORBI_3004G216900* (currently annotated as *SbMYB56*) displayed a similar directional shift during acclimation and was upregulated at 12 h and 24 h (log_2_FC_12h→24h_ = 4.22 → 3.98; padj_12h→24h_ = 3.34 × 10^−6^ → 1.34 × 10^−4^; kME_black = −0.91) (Supplementary Materials Table S5).

Although heat shock factors (HSFs) are primarily associated with heat stress, recent studies indicate that members of this family also participate in cold stress responses [55]. Specific HSF transcription factors in sorghum (e.g., *HSF1*, *HSF5*) have been predominantly linked to heat and drought stress in the literature [75]. RNA-seq analyses have reported high transcript accumulation of the heat shock protein gene *Sb03g027330* (*SORBI_3003G214000*; *SbHSP*) under cold exposure [6,60]. In the present study, this gene followed the tan module trend, being repressed during the initial shock phase but becoming significantly upregulated at 24 h, coinciding with the potential onset of acclimation (Supplementary Materials Table S5).

### 4.5. Post-Transcriptional Refinement: Alternative Splicing (AS)

Alternative splicing (AS) may act as a proactive adaptive control mechanism that potentially refines the cold-responsive transcriptome through isoform diversification [71]. In this dataset, AS events were detected in multiple transcription factor families, including *AP2/ERF-AP2* (*SORBI_3006G240700*), *NAC* (*SORBI_3006G281900*), *WRKY* (*SORBI_3009G034800*), and *SbB3-ARF*, across distinct stress phases (6 h, 12 h, and 24 h). Extensive AS regulation within these families has been reported under various abiotic stresses [50,76,77]. While *B3-ARF* genes show differential regulation under heat stress [75], the AS identified in *SbB3-ARF* in this study suggests a more nuanced regulatory layer. Beyond simple changes in transcript abundance, these AS-driven structural modifications are predicted to alter the C-terminal PB1 domain or the DNA-binding affinity of the protein, potentially influencing its interaction with Aux/IAA repressors [61,62]. Such isoform-level switching points to a possible fine-tuned modulation of auxin-associated growth pathways, which might serve as a molecular pivot to balance the growth–defense trade-off during cold acclimation.

A critical finding was the AS identification in signaling components implicated in the cold stress hierarchy. Notably, *SbCAMK_OST1L-2*, associated with stomatal regulation and the activation of the CBF pathway [55], exhibited an MXE-type splicing event at 12 h. This suggests that ABA-associated signaling is modulated at both transcript abundance and isoform levels. Similarly, SE-type splicing in the blue module hub *SbCAMK_CDPK-3* suggests post-transcriptional refinement of calcium signaling. This is consistent with findings in rice (*OsCPK13*, *15*, *18*, *19*) and *Vitis amurensis* (*VaCPK9*, *VaCPK3a*), where alternative splicing-generated isoforms are reported to contribute significantly to stress adaptation and growth regulation [78].

In contrast, data in this study suggest that while *PIF7* and *PIN3/SUF4* are crucial for temperature sensing, they were regulated exclusively at the transcriptional level [72,74] (Supplementary Materials Table S5). This indicates a functional partition, in which some systemic signals are controlled by transcript volume, while others, such as the MAPK cascade component *SbSTE_STE11-2*, utilize both SE- and MXE-type events. The simultaneous transcriptional induction and isoform switching of *SbSTE_STE11-2* at 24 h suggest a “dual-layer” regulatory mechanism. Similar to the functional specialization of *OsMAPK5a* in rice [79], the AS events in *SbSTE_STE11-2* have the potential to modify the kinase catalytic domain or protein-interaction surfaces, potentially contributing to the refinement of the MAPK signaling axis. The scope of AS regulation is further extended to epigenetic remodelers, including SbSNF2-2, SbSET-2, and SbIWS1-2. The SE-type event in *SbSET-2* at 12 h, preceding its strong induction at 24 h, suggests that epigenetic adjustment is a time-sensitive process. This observation is consistent with the hypothesis that chromatin marks (e.g., H3K36me3) may influence AS patterns during prolonged exposure [71]. Finally, the involvement of *SbeIF2α-1* via AS suggests that transcript-level diversification may intersect with known translational regulators, which could contribute to energy conservation during late-stage acclimation.

### 4.6. Transcriptional Signatures of Late Acclimation

At 24 h of cold stress, the transcriptional landscape exhibited reduced dynamic variation compared to earlier time points, indicating a transition toward a stabilized regulatory state rather than an absolute homeostatic equilibrium. During this final phase, the hormonal and functional transcriptional profile associated with acclimation is characterized by the coordinated expression patterns of members of the GRAS and HB-BELL families. Specifically, a DELLA protein within the GRAS family (*SORBI_3002G363700*) and a PAT1 subgroup member (*SORBI_3002G354900*, also known as *SbGRAS18*) displayed sustained transcriptional prominence at this stage, supporting their potential regulatory contribution to the late-phase response. In accordance with the literature, DELLA proteins are known to suppress gibberellin signaling to restrict growth, while the PAT1 subgroup has been reported to activate the Jasmonic Acid (JA) pathway to bolster defense [6,80]. Simultaneously, an *HB-BELL* transcription factor (*SORBI_3001G102300*) was highlighted, showing maintained expression at 24 h. Members of the HB-BELL family have been reported to interact with KNOX proteins and influence cytokinin/gibberellin balance, as previously described in related studies [72,81]. Furthermore, the persistent activity of *SbCAMK_CDPK-3* (*SORBI_3010G174500*) during this phase was determined in this study, suggesting a potential maintenance of calcium-dependent signaling at the transcript level during late acclimation. While CDPK-mediated regulation of aquaporins and membrane-associated processes has been documented in previous studies [82], such downstream targets were not directly examined in the present analysis.

The observed transcriptional profiles also suggest that epigenetic mechanisms may contribute to the stabilization of the late-phase response. SET-domain proteins are known to modulate chromatin accessibility through histone methylation at residues such as H3K4, H3K9, H3K27, and H3K36. In sorghum, 34 SET-domain proteins have been annotated, suggesting a diversified chromatin regulatory capacity. In the present dataset, histone methyltransferases *SbSET-1* (*SbSDG8*) and *SbSET-2* were induced during the late phase, which may point toward chromatin remodeling under cold stress. While the current analysis does not directly assess epigenetic marks, the transcriptional activation of these regulators is consistent with previous reports describing SET-domain proteins as contributors to stress-associated chromatin memory [65]. BR-dependent transcription factors such as *BZR1* and *BES1* have been reported to promote the expression of cryoprotective genes, thereby contributing to stress-associated stability [55]. The coordinated upregulation of *SbSNF2-1* and *SbIWS1-2* at the late stage hints at an integrated chromatin–transcriptional regulatory response in sorghum during cold acclimation. This pattern is consistent with previous reports describing the functional interplay between chromatin remodelers and transcription-associated factors [64].

### 4.7. Limitations and Future Perspectives

Compared to earlier transcriptomic studies in sorghum, which primarily focused on cataloging differentially expressed genes (DEGs) or single-gene families under cold stress [83], an advancement in understanding is provided through the integration of systems-level network modeling (WGCNA) with temporal alternative splicing (AS) dynamics. However, it is explicitly acknowledged that this study is fundamentally an integrative bioinformatic re-analysis and is intended to serve as a hypothesis-generating framework rather than a functionally validated endpoint. In recent state-of-the-art crop cold-stress studies, such as advanced pan-transcriptomic analyses in rice, long-read sequencing technologies (e.g., Iso-Seq) have been employed to resolve full-length isoform structures, and experimental models (e.g., CRISPR/Cas9 mutants or isoform-specific RT-PCR) have been used to confirm the causal, mechanistic roles of specific splicing factors [50]. While such a level of mechanistic validation is not achieved in the present sorghum analysis, and the interpretation relies on homology-based functional annotations, this study helps bridge the gap between basic DEG profiling and advanced functional genomics. By prioritizing high-confidence, temporally regulated hub genes and specific AS events, a robust and prioritized roadmap for future targeted experimental validation in sorghum is provided.

## 5. Conclusions

This study describes a phase-dependent organization of the cold stress response in sorghum, characterized by coordinated metabolic, regulatory, and transcriptomic transitions over time. The early shock phase (6 h) is marked by the broad repression of transcripts associated with energy metabolism and organelle function, alongside a temporary reduction in cell surface receptor signaling. Together, these transcriptomic shifts point toward a potential intracellular prioritization of energy conservation and stress signal processing over active growth.

During the transition to acclimation (12 h), the sustained activation of core transcription factor families (e.g., AP2/ERF and NAC) maintains the central cold-responsive regulatory circuit. In parallel, transcriptional signatures suggestive of translational attenuation and the progressive upregulation of chromatin-associated remodelers imply a potential transition toward more stabilized, epigenetically supported regulatory states. This phase reflects a shift from rapid stabilization to regulatory consolidation.

The late acclimation phase (24 h) is defined by complex regulatory inversions and the persistent activity of calcium-dependent and MAPK-associated signaling components. This suggests the establishment of a new homeostatic balance, potentially integrating sustained signaling with longer-term structural and chromatin-level adaptations. Furthermore, the extensive involvement of alternative splicing across signaling kinases, transcription factors, and epigenetic regulators establishes isoform diversification as a crucial parallel regulatory layer operating alongside differential expression.

Collectively, the integration of differential expression, network topology, and alternative splicing across temporal phases provides a comprehensive, phase-dependent working model of sorghum cold acclimation. However, as a fundamentally bioinformatical framework, the inferred regulatory mechanisms proposed here—particularly regarding translational control and epigenetic stabilization—require future targeted experimental validation (e.g., isoform-specific RT-PCR, mutant analysis, or chromatin profiling) to definitively confirm their functional roles in C4 crop resilience.

## Data Availability

The transcriptomic dataset analyzed in this study corresponds to the cold stress subset of accession number PRJNA1159475, publicly available in the National Center for Biotechnology Information (NCBI) Sequence Read Archive (SRA) database (https://www.ncbi.nlm.nih.gov/sra; retrieved on 26 January 2026).

## Supplementary Materials

The following supporting information can be downloaded at: https://www.mdpi.com/article/10.3390/biology15070560/s1, Figure S1: Scale-free topology fit index and mean connectivity plots for soft-threshold power selection. Figure S2: Gene dendrogram and module assignments. Figure S3: Module–trait relationships. Figure S4: Co-expression network of the green module highlighting the strong connectivity of *SORBI_3003G396600* with *SORBI_3006G143200* (RNA helicase) and *SORBI_3002G234300* (nucleolar protein). Figure S5: Network topology illustrating the connectivity patterns of *SORBI_3003G282000* (*SbeIF2α-1*; *eIF2α* homolog) and related homologs within the co-expression network. Table S1: Representative distribution of major TF families across time points (6, 12, and 24 h) and their intersections (filtered by adj *p* < 0.01). Table S2: Topological hub genes in WGCNA. Table S3: High-confidence transcription factors (TFs) associated with the cold stress response in sorghum. Table S4: High-confidence TRs and PKs associated with the cold stress response in sorghum. Table S5: Expression profiles of previously reported cold-responsive genes in sorghum.

## Abbreviations

The following abbreviations are used in this manuscript:

A3SS	Alternative 3′ splice site
A5SS	Alternative 5′ splice site
ABA	Abscisic acid
ADK	Adenylate kinase
AP2/ERF	APETALA2/Ethylene Responsive Factor
AS	Alternative splicing
bp	Base pair
CBF	C-repeat binding factor
CDPK	Calcium-dependent protein kinase
COR	Cold-regulated genes
COX6A	Cytochrome c oxidase VIa
DAS	Differential alternative splicing
DEG	Differentially expressed gene
DREB	Dehydration-responsive element-binding
FDR	False discovery rate
GO	Gene Ontology
IGV	Integrative Genomics Viewer
kME	Intramodular connectivity
log2FC	Log2 fold change
MAPK	Mitogen-activated protein kinase
ME	Module eigengene
MXE	Mutually exclusive exons
NAC	NAM, ATAF, and CUC transcription factor
NCBI	National Center for Biotechnology Information
padj	Adjusted *p*-value
PCA	Principal Component Analysis
PK	Protein kinase
PPR	Pentatricopeptide repeat
PSI	Percent Spliced In
RI	Retained intron
RNA-Seq	RNA sequencing
ROS	Reactive oxygen species
SE	Skipped exon
SRA	Sequence Read Archive
TF	Transcription factor
TOM	Topological overlap matrix
TPT	Triose phosphate transporter
TR	Transcriptional regulator
VST	Variance Stabilizing Transformation
WGCNA	Weighted Gene Co-expression Network Analysis
